# Recovery of the first full-length genome sequence of a parapoxvirus directly from a clinical sample

**DOI:** 10.1038/s41598-017-03997-y

**Published:** 2017-06-16

**Authors:** Thomas Günther, Ludwig Haas, Malik Alawi, Peter Wohlsein, Jerzy Marks, Adam Grundhoff, Paul Becher, Nicole Fischer

**Affiliations:** 1 Heinrich-Pette Institute, Leibniz Institute for Experimental Virology, Hamburg, Germany; 2 Institute of Virology, University of Veterinary Medicine, Hannover, Germany; 3 Bioinformatics Core, University Medical Center Hamburg-Eppendorf, Hamburg, Germany; 4 Department of Pathology, University of Veterinary Medicine, Hannover, Germany; 5 Profesor Krzysztof Skóra Hel Marine Station, Institute of Oceanography, University of Gdańsk, Gdańsk, Poland; 6 German Center for Infection Research, Hamburg – Borstel – Lübeck – Riems, Germany; 7 German Center for Infection Research, Hannover – Braunschweig, Germany; 8 Institute for Medical Microbiology, Virology and Hygiene, University Medical Center Hamburg-Eppendorf, Hamburg, Germany

## Abstract

We recovered the first full-length poxvirus genome, including the terminal hairpin region, directly from complex clinical material using a combination of second generation short read and third generation nanopore sequencing technologies. The complete viral genome sequence was directly recovered from a skin lesion of a grey seal thereby preventing sequence changes due to *in vitro* passaging of the virus. Subsequent analysis of the proteins encoded by this virus identified genes specific for skin adaptation and pathogenesis of parapoxviruses. These data warrant the classification of seal parapoxvirus, tentatively designated SePPV, as a new species within the genus *Parapoxvirus*.

## Introduction

Parapoxviruses (PPVs) form a genus of the family *Poxviridae*. Poxviruses are large double stranded DNA viruses with genomes of approximately 135 to 360 kbp, which contain up to 328 open reading frames^1^. According to the International Committee on Taxonomy of Viruses (ICTV; http://ictvonline.org/virusTaxonomy.asp), the genus *Parapoxvirus* comprises the following species members: the Orf virus (ORFV), considered the prototype parapoxvirus causing contagious pustular dermatitis in small ruminants, the Bovine papular stomatitis virus (BPSV), the Pseudocowpox virus (PCPV) and the Parapoxvirus of red deer in New Zealand (PVNZ). Beside these viruses with known full-length nucleotide sequences, a number of tentative species have been proposed based on the amplification of smaller genome fragments using pan-PCR primers encompassing 250–550 bp of the DNA polymerase, DNA topoisomerase I and major envelope protein encoding regions. These species comprise reindeer musk ox parapoxviruses^2, 3^, cattle parapoxviruses^4^, pinniped parapoxviruses^5^ and a very recently described, putative novel parapoxvirus in horses^6^. PPVs are considered as zoonotic and can cause circumscribed skin lesions in humans, historically best known as milker’s nodules. Typical lesions of this type have also been described in humans who have come in contact with PPV infected seals^7, 8^.

Parapoxvirus infections have been reported in different seal species and sea lions of the Atlantic and Pacific oceans including habitats in the sub-arctic, arctic, and antarctic waters^5, 9–16^. They typically cause pustular skin lesions and ulcerations around the mouth and on the flippers of the animals as well as mucosal lesions with ulcerations in the oral cavity. Parapoxvirus infections are diagnosed by clinical evaluation of skin and mucosal lesions together with electron microscopy or immunohistochemistry analyses, isolation of viral particles and/or detection of viral sequences by polymerase chain reaction (PCR). Infections are generally self-limiting after 1–6 weeks, but in the case of bacterial superinfections they may result in severe ulcerative and necrotizing lesions. Since the full-length genome sequence of seal parapoxvirus had previously not been determined, classification of the virus was derived from short sequence fragments amplified from infected tissues^5, 7–10, 12–19^. Based on phylogenetic analysis of these sequences, it was suggested that the parapoxviruses of seals may belong to a separate species within the genus *Parapoxvirus* ^5^.

Here, we report a clinical case of a PPV infection in a grey seal (*Halichoerus grypus*) found in the Baltic Sea in Poland in April 2015. Primary laboratory diagnosis was based on histologic evaluation and *in situ* hybridization, as well as electron microscopy and PCR analysis. Next generation sequencing (NGS) was performed on DNA extracted directly from skin lesion material. We employed a combination of second (Illumina MiSeq) and third (Oxford Nanopore MinION^20^) generation sequencing to recover the full-length genome of this seal parapoxvirus, including telomere sequences and hairpin structure. To our knowledge, this is the first report of a full-length seal parapoxvirus genome sequence, as well as the first report of recovery of a full-length parapoxvirus sequence directly from clinical material. Thus, the full-length genome sequence reported here can be assumed to faithfully reflect the naturally acquired pathogenic parapoxvirus strain, without any adaptions resulting from *in vitro* culturing^18, 21^.

## Results and Discussion

### Identification of a parapoxvirus from a grey seal (Halichoerus grypus)

**Figure 1:**
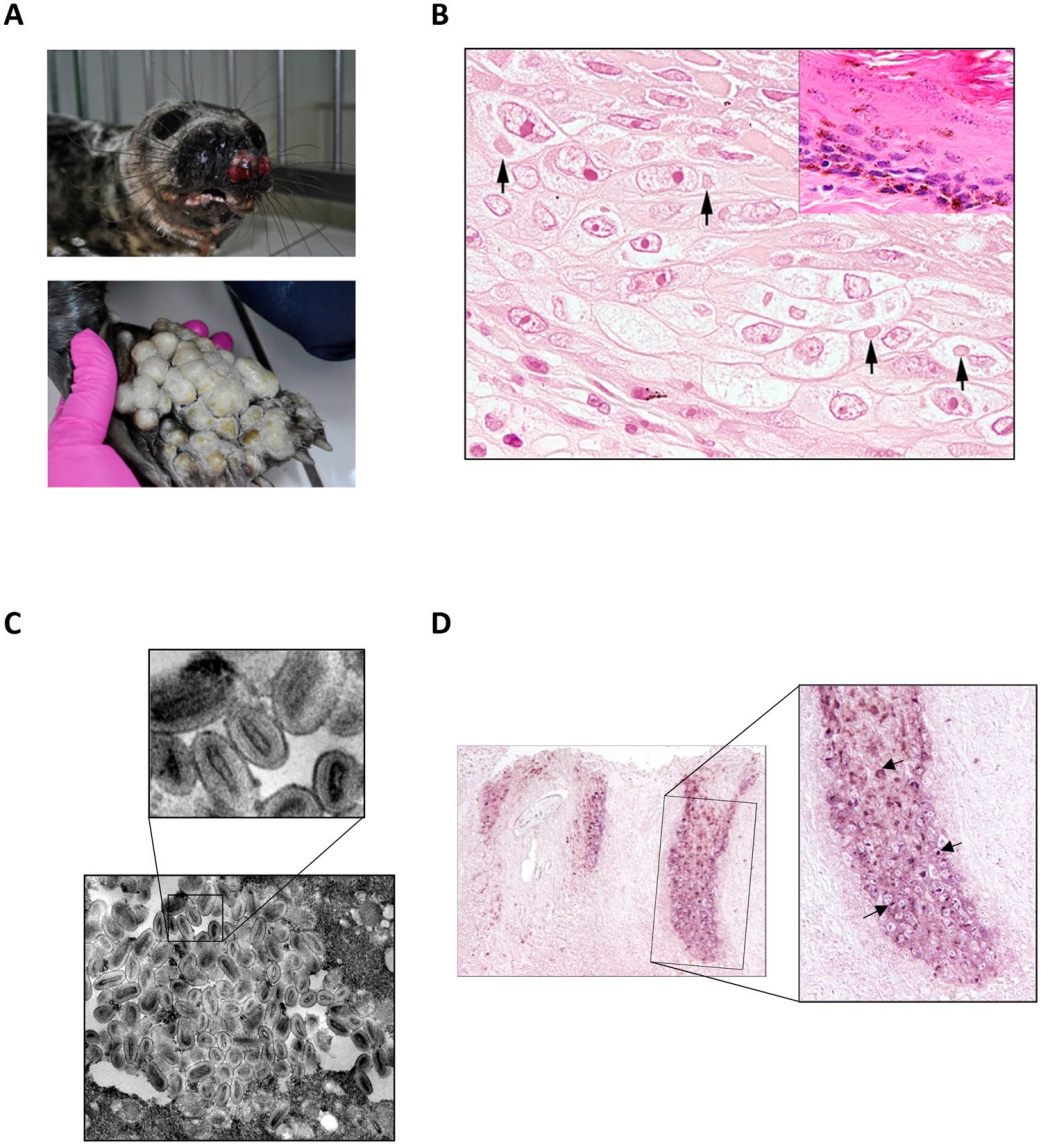
Macroscopic (**A**), electronmicroscopical (**B**) and histological analysis (**C**) of a harbor seal infected with seal parapoxvirus. (**A**) Ulcerative nodular skin lesion were identified on both front fins and the muzzle of the animal. (**B**) Histological changes of hair follicle epithelium characterized by ballooning degeneration and cytoplasmic eosinophilic inclusion bodies (arrows), magnification 600x. Normal hair follicle epithelium of an unaffected grey seal is shown in the upper right corner, HE, magnification 600x. (**C**) Electron microscopy pictures of the cytoplasm of a hair follicle epithelial cell isolated from an infected seal skin lesion. Magnification 37,500x. Mature and immature virus like particles were densely packed. The ovoid to lancealate shape of the core virions are clearly visible. (**D**) *In situ* hybridization using a digoxigenin-labeled parapoxvirus-specific DNA probe: parapox virus specific signal is detected in hair follicle epithelial cells of the suprabasal layers (arrow), while no specific signal was detected in the basal layers. Left panel represents an overview with a 100 x magnification, right panel shows a blown up of the highlighted area, magnification: 200x.

In April 2015, a young grey seal taken to a seal rehabilitation center showed large spherical dermal lesions with severe ulcerations at both flippers and in the mucosa of the oral cavity (Figure 1A). Histological examination of the affected skin areas showed extensive ulcerations and necrosis with epidermal loss and infiltration of neutrophils and few macrophages. Ballooning degeneration with severe swelling of hair follicular epithelial cells was observed. Occasionally, cytoplasmic eosinophilic inclusion bodies were present (Figure 1B). Transmission electron microscopical analysis of follicular epithelial cells clearly identified densely packed, multifocal clusters of viral particles in the cytoplasm (Figure 1C) indicative of parapoxvirus particles based on the elongated, ovoid shaped core surrounded by a membrane and a superficial membrane. Enveloped particles had a length of approximately 200 to 250 nm and a width of 100 to 150 nm.

Parapoxvirus infection was confirmed by performing a pan-parapoxvirus PCR targeting a 552 bp sequence of the genomic region encoding the major envelope protein^5^. Sequence analysis established 97.3% homology to the partial seal parapoxvirus (SePPV) sequences described earlier^5^. *In situ*-hybridization (ISH) with a probe specific for the amplified region revealed abundant parapoxvirus DNA–positive hair follicle epithelial cells corresponding to the cells with ballooning degeneration (Figure 1D). In contrast, cells of the basal cell layer lacked a specific hybridization signal (Figure 1D).

Unfortunately, virus isolation using a seal tissue-derived cell line was not successful. Limiting amount of tissue and suboptimal condition of the tissue most likely contributed to the toxic effects observed during cultivation processes.

### Recovery of the full-length seal parapoxvirus genome applying high throughput short read sequencing together with Nanopore MinION sequencing

DNA from skin lesion was subjected to high throughput multiplex sequencing on an Illumina MiSeq Instrument, as well as nanopore sequencing on a third generation Oxford Nanopore MinION device^20^. Approximately 5% out of a total of 2,272,653 short reads generated on the MiSeq instrument did not map to host sequences and thus were considered to be of potential exogenous origin. De novo assembly and iterative mapping of the non-host reads yielded 19 contigs (sizes between 590 bp and 23,767 bp) that showed distant sequence similarity to the virus family *Poxviridae*, genus *Parapoxvirus*. These contigs accounted for 68.33% (71,680 reads) of all non-host reads; there were no other contigs or reads indicative of the presence of other pathogenic viruses in this sample. Iterative mapping of all sequences to full-length genome of ORFV allowed the assembly of a single contig of 127,941 bp (minimal coverage: 260 over 99% of the contig) (Table 1). The sequence was classified as a seal parapoxvirus due to its close homology to a short fragment from the major envelope protein-coding sequence described in 2002^5^. We used nanopore sequencing to verify the assembly of short sequencing reads along the coding region and the termini of the virus. Nanopore sequencing produces relatively high error rates and thus is of limited use for de novo sequencing. However, the long read lengths which can be produced by this technique make it ideally suited to confirm the overall structure of sequence contigs. As shown in Figure S1 and Table S1, nanopore sequencing of the primary clinical material produced a total of 48 reads which mapped across at least 30 kbp of the viral genome, with the longest read covering a continuous stretch of 56.2 kbp. Together, although there were a few gaps at the right end of the genome, the nanopore reads covered more than 92% of the assembled genome, thus confirming the accuracy of the short read assembly.

**Table 1:** Summary of size, relative GC content and number of open reading frames in all full-length genomes of the genus parapoxvirus.

Name	Genbank accession number	Size (bp)	GC content (%)	number of annotated ORFs
Orf Virus (ORFV)	AY386264.1	139,981	65.0	134
Seal Parapoxvirus (SePPV)	KY382358	127,941	55.9	119
Red Deer Parapoxvirus (PVNZ)	NC_025963.1	139,962	63.4	132
Bovine papular stomatitis virus (BPSV)	NC_005337.1	134,431	64.5	134
Pseudocowpoxvirus (PCPV)	NC_013804.1	145,289	65.0	134

**Figure 2:**
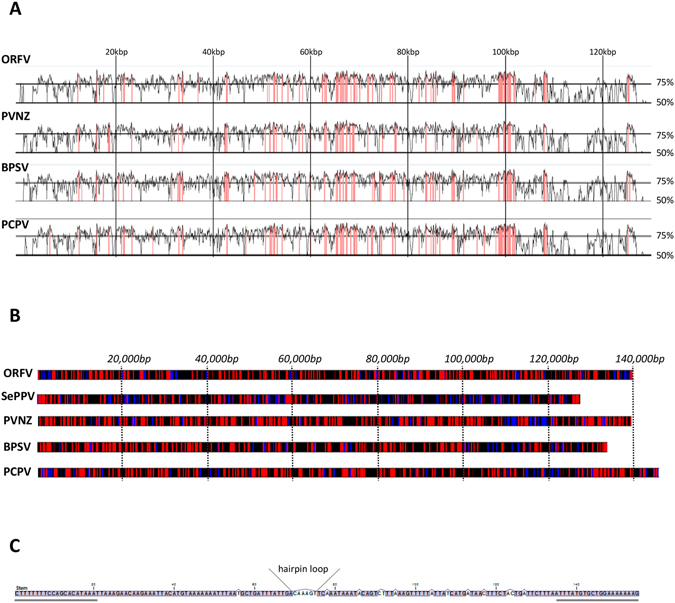
Genome Characterization of the full-length genome seal parapoxvirus sequence. (**A**) Sequence alignment of seal parapoxvirus (KY382358) to all four reference genomes (ORFV: AY386264.1, PVNZ: NC_025963.1, BPSV: NC_005337.1, PCPV: NC_013804.1) in the genus parapoxvirus. The alignment was performed using the global alignment program AVID implemented in VISTA (tool for comparative genomics). Alignments were visualized with VISTA point (Calc Window, bp: 100; Min Cons Width, bp: 100; Cons Identity, %: 90 Minimum Y, %:50). The graph represents percent conservation between the aligned sequences at a given coordinate on the base sequence. Highly conserved regions, with a conservation higher than 90%, are shown in pink. (**B**) G + C genome profile of all reference genomes (AY386264.1, NC_025963.1, NC_005337.1, NC_013804.1) listed in Genbank for parapoxviruses together with the newly identified seal parapoxvirus. Each trace represents the % G + C content of the indicated viral genome. GC content is indicated by the color scheme with blue representing a GC content range from 0–33.3%, black from 33.3–66.6% and red from 66.6–100%. (**C**) Terminal hairpin sequences of the seal parapoxvirus genome. SePPV hairpin terminus consists of an incomplementary base-paired and AT rich sequence. Telomere resolution sequence is underlined.

Nucleotide sequence alignments among all fully sequenced parapoxvirus genomes revealed that the seal virus is only distantly related to the other genus members, showing the closest homology (77.3% sequence identity) to the bovine papular stomatitis virus (BPSV) sequence (Figure 2A, Table 2). The seal parapoxvirus, tentatively named SePPV, has the smallest genome (128 kbp) among the fully sequenced members within this genus, followed by bovine papular stomatitis virus with 138 kbp (Figure 2B). Similar to other parapoxviruses, the SePPV genome sequence shows a relatively high GC content. However, with 55.9% the GC content is the lowest known so far within this genus (Figure 2B; Table 1). In addition to the core sequence, we were able to resolve the hairpin termini of the parapoxvirus genome, including the telomere resolution sequence important for effective replication of the virus (Figure 2C). The inverted terminal repeats (ITR) of SePPV encompass 2,087 bp, coordinates 1–2,087 sense orientation and 125,855–127,941 antisense orientation. Interestingly the ITR contains a complete duplication of the ORF encoding for a dUTPase (similar to ORF007 dUTPase, Supplementary Dataset S1). In addition, the ITR contains a partial duplication of the ankyrin repeat (similar to ORF008 ankyrin repeat, Supplementary Dataset S1). While a partial duplication of an ankyrin repeat has been described for Camelpox virus (AF438165), the observed duplication of the ORF007 has not been described for parapoxviruses or poxviruses in general.

**Table 2:** Percentage nucleotide identity between the full-length genome sequences within the genus parapoxvirus.

	SePPV	ORFV	BPSV	PCPV	PVNZ
SePPV	100	76.9	77.3	77.1	76.0
ORFV	76.9	100	81.2	**90.9**	81.0
BPSV	77.3	81.2	100	82.0	81.0
PCPV	77.1	**90.9**	82.0	100	81.5
PVNZ	76.0	81.0	81.0	81.5	100

SePPV (KY382358); ORFV (AY386264.1); PVNZ (NC_025963.1); BPSV (NC_005337.1); PCPV (NC_013804.1).

The contig generated by de novo assembly contained 230 single open reading frames (ORFs) of which 120 were identified as putative SePPV genes. Of these, 116 ORFs are coding for proteins with significant homology to annotated ORFs in other parapoxviruses (Table 1; Supplementary Datasets S1, S2). The relative order of the genes is similar to other parapoxviruses thereby supporting the classification as a novel species within the genus *Parapoxvirus*. Similar to the other members of the genus *Parapoxvirus*, the SePPV contains ORFs encoding for proteins involved in pathogenesis (Supplementary Datasets S1, S2). SePPV encodes a viral homologue of IL10 (SePPVgORF114), which has been shown in ORFV to be a potent anti-inflammatory virokine since deletion of the ORF results in an attenuated virus^22, 23^. In addition, SePPVgORF101 expresses an anti-inflammatory chemokine binding protein CBP, which in ORFV plays a role in disrupting chemotactic recruitment of leukocytes^24^. SePPV contains an ORF, SePPVgORF013, of which the gene product has significant homology to an inhibitor of interferon response which blocks activation of the dsRNA dependent protein kinase^25^. SePPV also encodes proteins, encoded by SePPVgORF017 and SePPVgORF109 with significant homology to factors described for other parapoxviruses involved in NFκB signaling, ORF24 and ORF121. The function of the ORF24 gene product in ORFV is described to decrease TNFα induced phosphorylation whereas ORF121 of ORFV most likely is involved in the inhibition of NFκB-p65 phosphorylation and nuclear translocation^26, 27^. As described for all established parapoxvirus species, we also identified an ORF encoding a vascular endothelial growth factor (VEGF) homologue^28–31^. The SePPVgORF118 encoded VEGF shows closest homology to BPSV, however different to BPSV with regard to the location of the ORF coding for VEGF, SePPV VEGF is located at the right end of the genome^29, 32^. Viral VEGF most likely enhances viral growth by promoting cellular regeneration of the epidermis. Viruses devoid of VEGF do not induce extensive blood vessel formation and dermal swelling which is discussed to provide protection for immune cells^24^. Interestingly, BPSV encoded VEGF different to all other parapoxvirus encoded VEGFs shows higher homology to mammalian VEGF-A^24, 29^.

SePPV encodes for 4 unique open reading frames not identified in ORFV and other parapoxviruses. The hypothetical proteins encoded by these ORFs do not show any significant homology with known proteins from the family *Poxviridae* (Supplementary Dataset S1). In comparison to ORFV, 16 ORFs including 9 hypothetical proteins, 2 ankyrin repeat proteins, 2 putative IMV membrane proteins and 2 proteins involved in virion morphogenesis are not present in SePPV (Supplementary Table S2). In addition, different to ORFV and other parapoxviruses we did not identify inhibitors of granulocyte-macrophage colony stimulating factor and interleukin-2, which are known as GIF (Supplementary Table S2) and play a role in the regulation of the adaptive immune response of the host^33, 34^.

As shown in Tables 3 and 4, on the protein level SePPV demonstrates relatively uniform distances to other members of the genus (84.3 and 84.5% mean amino acid identity for DNA polymerase and DNA topoisomerase, respectively), with the closest relative again being BPSV.

**Table 3:** Percentage amino acid identity between the DNA polymerase within the genus parapoxvirus.

	SePPV	ORFV	BPSV	PCPV	PVNZ
SePPV	100	84.08	84.77	83.78	84.57
ORFV	0.17	100	86.72	**94.05**	85.43
BPSV	0.16	0.14	100	88.11	87.12
PCPV	0.17	0.06	0.13	100	85.53
PVNZ	0.17	0.16	0.14	0.16	100

Upper comparison gradient indicates percentage identity between two sequences; lower comparison gradient indicates the distance between two sequences as calculated by the distance measure Jukes-Cantor. Percentage identity higher than 90% is shown in bold numbers.

**Table 4:** Percentage amino acid identity between the DNA topoisomerase I within the genus parapoxvirus.

	SePPV	ORFV	BPSV	PCPV	PVNZ
SePPV	100	83.96	86.79	83.65	83.44
ORFV	0.18	100	86.79	**95.91**	83.75
BPSV	0.14	0.14	100	88.36	84.69
PCPV	0.18	0.04	0.12	100	84.06
PVNZ	0.18	0.17	0.16	0.17	100

Upper comparison gradient indicates percentage identity between two sequences; lower comparison gradient indicates the distance between two sequences as calculated by the distance measure Jukes-Cantor. Percentage identity higher than 90% is shown in bold numbers.

**Figure 3:**
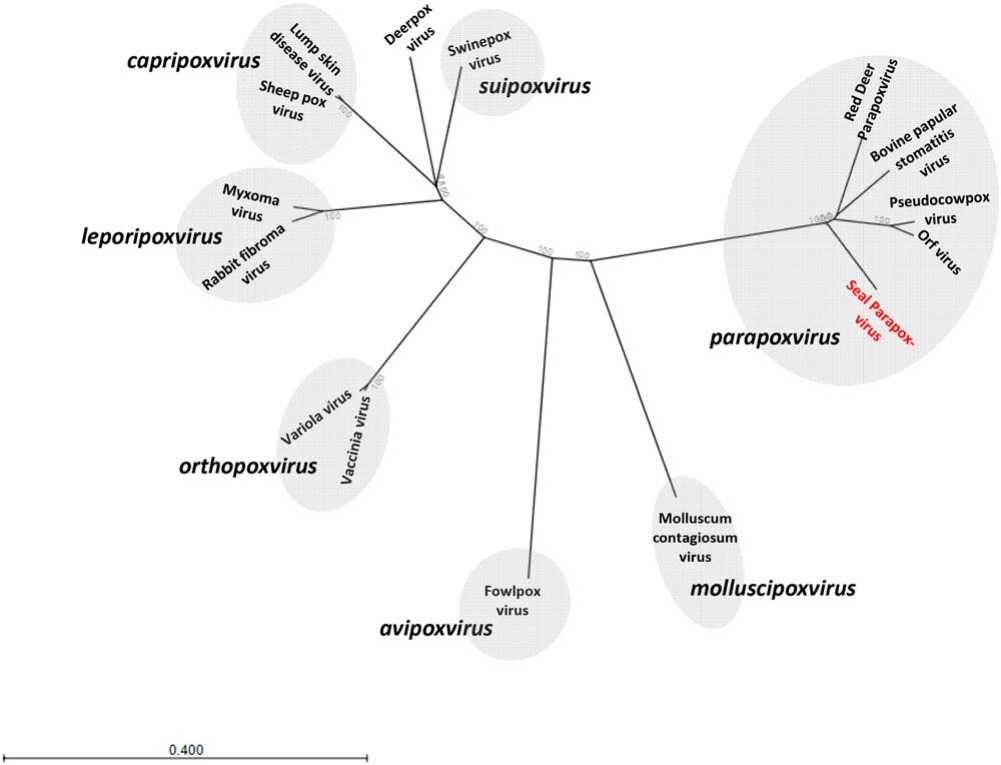
Phylogenetic tree analysis based on 47 proteins. Protein sequences were considered being conserved, if the corresponding sequence of SePPV yielded BLASTP alignments over at least 90% of the SePPV protein sequence length with sequences of all 14 representative genomes. The following sequences were used: Red Deer Parapoxvirus (PVNZ) HL953 (NC_025963.1); ORFV (AY386264.1); PCPV (NC_013804.1); BPSV (NC_005337.1); Vaccinia Virus (NC_006998.1); Variola Virus (NC_001611.1); Myxoma Virus (NC_001132.2); swinepox virus (NC_003389.1); deerpox virus (NC_006966.1); sheep pox virus (NC_004002.1); lumpy skin disease virus (NC_003027.1); fowlpox virus (NC_002188.1); rabbit fibroma virus (NC_001266.1); molluscum contagiosum virus (NC_001731.1).

For phylogenetic analysis all proteins in SePPV sequence were aligned with proteins identified in 14 representative genomes of the subfamily Chordopoxvirinae. Proteins which showed an alignment of >90% of the length of the individual protein within SePPV were used to construct a concatenated polyprotein. This polyprotein consisting of 47 proteins was used for the phylogenetic tree analysis (Figure 3). In addition, phylogenetic analysis was also applied with complete coding sequences of the DNA polymerase and DNA Topoisomerase I confirming the results obtained with the concatenated polyprotein (supplementary material Figure S2A,B, Tables 3 and 4). Thus, the results of our phylogenetic tree analyses together with the description of the gene order and annotated ORFs clearly warrant the classification of SePPV as a distinct species within the genus *Parapoxvirus*.

## Conclusion

We report the first reconstruction of a full-length genome sequence of a member of the family *Poxviridae* directly from a clinical sample using Illumina short read sequencing combined with Oxford Nanopore sequencing. Poxviruses are challenging to grow in culture and culture adapted genomes might not faithfully represent those of virulent field strains. The power of combining high throughput short read sequencing with nanopore sequencing to recover a large and complex viral genome directly from complex clinical material as demonstrated here should be useful also for other virus families in particular those with large viral genomes.

## Methods

### Clinical case

In April 2015, a young grey seal was taken to the Hel Marine Station, a field station belonging to the Institute of Oceanography at the University of Gdansk, Poland. A few days later, skin lesions appeared on the front flippers followed by lesions around mouth and nose (Figure 1A). Skin samples from lesions of the left front flipper were sent to the University of Veterinary Medicine, Hannover, Germany, for further diagnosis in the framework of veterinary microbiological diagnostics in accordance to the German legislation. Therefore no ethical approval was required for the use of these samples. Upon symptomatic treatment, the lesions declined and the seal could eventually be discharged in June 2015.

### Polymerase chain reaction (PCR) and Sanger Sequencing

Total DNA was extracted from skin lesions using a High Pure PCR Template Preparation Kit (Roche Diagnostics).

Conventional PCR was done as described before^5^, amplifying a part of the putative major envelope gene. To confirm the specificity of PCR, amplicons were sequenced (LGC Genomics GmbH, Berlin, Germany) and aligned using the Clustal W multiple alignment tool implemented in BioEdit^35^.

### Histology and *in-situ* hybridization

Formalin-fixed tissue biopsies of the altered skin were processed routinely into paraffin wax. Tissue section of 3–4 µm thickness were cut and stained with hematoxylin and eosin (HE) for light microscopical examination.

*In situ*-hybridization was performed as previously described using an universal parapox primer pair, P1 (5′-GTCGTCCACGATGAGCAGCT-3′) and P2 (5′-TACGTGGGAAGCGCCTCGCT-3′; GeneBank accession number U06671), and a digoxigenin-labeled DNA probe^19^.

For transmission electron microscopy a direct pop-off technique from the HE-stained slide was used as previously described^36^.

### Illumina library preparation and MiSeq short read sequencing

Total DNA from 75 mg tissue was mechanically homogenized in PBS using disposable tissue grinder pestles. After low speed pelleting, supernatant was filtered (0.2 µm) and DNA was subsequently isolated using DNeasy blood & tissue Kit (Qiagen).

DNASeq library compatible with short read Illumina sequencing was generated using the NEB Ultra DNA library Kit (NEB) starting with 500 ng DNA, as measured by Qubit (Invitrogen) and following the manufacturer’s instructions. Briefly, DNA was fragmented, end repaired and subsequently the adapter were ligated. Agencourt AMPure XP beads were used to size select the DNA fragments containing the adapters. Finally, the library was amplified by 15 PCR cycles. The fragment size distribution of the library was analyzed on a BioAnalyzer High Sensitivity LabChip showing a size range between 400 and 446 bp with the main peak of the library at 401 bp. The library was diluted to 2 nM and multiplex-sequenced together with five samples on the Illumina MiSeq (2 × 250 bp paired end run, estimated 4.3 million reads/sample).

### Oxford Nanopore library preparation and MinION sequencing

Nanopore sequencing library preparations using Nanopore Sequencing Kits SQK-MAP005 and SQK-MAP006 were essentially performed as described in the protocols and guidelines provided by Oxford Nanopore Technologies (ONT). Briefly, 1 µg of the genomic DNA isolated from skin lesions was fragmented to an average size of 8–15 kb using g-TUBEs (Covaris). DNA fragments were end-repaired and adenylated using NEBNext Ultra II End-Repair/dA-tailing Module (NEB) followed by cleanup with Ampure XP beads (Beckmann Coulter). Sequencing and hairpin adapters (ONT) were ligated using NEB Blunt/TA Ligase Master Mix (NEB) followed by incubation with the hairpin tether (ONT). Cleanup of libraries was done either with His-Tag beads or MyOne C1 streptavidin beads (Invitrogen) depending on the respective Sequencing Kit and flowcell version. Prepared libraries were eluted in 25 µl of the ONT-supplied elution buffer.

Prior to sequencing, 6 μl of the eluate (pre-sequencing mix), 75 μl running buffer (ONT), 60 µl nuclease free H2O and 4 μl fuel mix (ONT) were combined gently and were immediately loaded onto the prepared MinION flowcells. Sequencing was performed using 48 hr sequencing run scripts with addition of freshly prepared input material to the MinION flowcell every 12 hrs until no further active pores were available anymore.

### Sequence assembly

Illumina reads were aligned to the Leptonychotes weddellii (Wedell seal) reference assembly (GCF_000349705.1) using Bowtie2 (v2.2.3)^24^. Reads yielding significant alignments with the reference assembly were excluded from further analysis. The remaining short reads were initially assembled into contigs using Trinity (r20140717).

Nanopore events were converted into FastQ containing Fast5 data using Metrichor basecalling with the respective 2D workflows. Fasta files were extracted from Fast5 data using poretools^25^. Long reads (>3000 bp) were subsequently aligned with LAST^26^ to the SePPV assembly generated from Illumina sequencing data using the following parameters: –s2 –T0 –Q0 –a1 –f1. Alignments were further filtered by a minimum length of 3,000 bp to reduce false positive results due to low complexity region alignment. The joint assembly of MinION and MiSeq was performed using SPAdes (v3.6.0) using the ‘careful’ option and otherwise standard parameters^37^.

### Phylogenetic analysis

Nucleotide sequences of viruses classified to the genus *Parapoxvirus* or to the family of *Poxviridae* were downloaded from GenBank: Red Deer Parapoxvirus (PVNZ) HL953 (NC_025963.1); ORFV (AY386264.1); PCPV (NC_013804.1); BPSV (NC_005337.1); Vaccinia Virus (NC_006998.1); Variola Virus (NC_001611.1); Myxoma Virus (NC_001132.2); swinepox virus (NC_003389.1); deerpox virus (NC_006966.1); sheeppox virus (NC_004002.1); lumpy skin disease virus (NC_003027.1); fowlpox virus (NC_002188.1); rabbit fibroma virus (NC_001266.1), molluscum contagiosum virus (NC_001731.1).

Amino acid sequences of single proteins (DNA polymerase and DNA topoisomerase I) were aligned using the CLUSTAL W multiple alignment tool, CLC Main workbench, version 7.6.4. For phylogenetic analyses, genomes were trimmed manually and neighbor-joining trees were calculated using nucleotide distance measurement Jukes-Cantor parameters. Bootstrap analysis was performed with 1000 iterations.

Following an approach described before^38^ phylogenetic analysis of a concatenated protein sequences was performed by maximum-likelihood tree construction using PHYML3^39^ (Figure 3). The sequences were obtained by identifying 47 proteins conserved in SePPV and 14 representative genomes (AY386264; NC_001132.2; NC_001266; NC_001611.1; NC_002188.1; NC_003027.1; NC_003389.1; NC_004002.1; NC_005337.1; NC_006966.1; NC_006998.1; NC_013804.1; NC_025963.1; NC_001731.1) of the subfamily Chordopoxvirinae. Protein sequences were considered being conserved, if the corresponding sequence of SePPV yielded BLASTP alignments over at least 90% of the SePPV protein sequence length with sequences of all 13 representative genomes.

## Electronic supplementary material

supplementary Material

Dataset 1

Dataset 2
