# Supplementary material for: Recovery of the first full-length genome sequence of a parapoxvirus directly from a clinical sample
Source: Sci Rep. 2017 Jun 16;7:3734. doi: 10.1038/s41598-017-03997-y (PMC5473882; doi:10.1038/s41598-017-03997-y)
Supplement: Supplementary file 1 — supplementary Material [file 41598_2017_3997_MOESM1_ESM.doc]

**Supplementary Material**

**Recovery of the first full-length genome sequence of a parapoxvirus directly from a clinical sample.**

Thomas Günther, Ludwig Haas, Malik Alawi, Peter Wohlsein, Jerzy Marks, Adam Grundhoff, Paul Becher, Nicole Fischer

**Supplementary Figure S1:** **MinION nanopore sequencing complements the Illumina assembly.**

1. Coverage of short reads generated by Illumina sequencing (upper panel) and reads longer than 3,000 bp generated by MinION sequencing. MinION long reads covered most parts of the assembly including the repeat region located at the left end arm of the SePPV genome. (B) The viral genome is indicated by a gray bar, including the identical repeats at the left and right end of the genome (dotted boxes). Minion reads are indicated by black bars.

**Supplementary Figure S2:** **Phylogenetic analysis of the complete coding region of the DNA polymerase (A) and DNA Topoisomerase I (B) of representative full-****length sequences within the different genera of the family *Poxviridae*.**

Phylogenetic trees were constructed by aligning the coding sequences using CLC Main workbench, version 7.6.4. Trimmed alignments were used for phylogenetic tree construction in CLC using the tree construction method neighbor-joining, nucleotide distance measurement Jukes-Cantor. Bootstrap analysis values are indicated as percentages. The tree is shown as a radial cladogram. Genbank accession numbers of the following sequences have been used in the alignment: Red Deer Parapoxvirus (PVNZ) HL953 (NC_025963.1); ORFV (AY386264.1); PCPV (NC_013804.1); BPSV (NC_005337.1); Vaccinia Virus (NC_006998.1); Variola Virus (NC_001611.1); Myxoma Virus (NC_001132.2); swinepox virus (NC_003389.1); deerpox virus (NC_006966.1); sheep pox virus (NC_004002.1); lumpy skin disease virus (NC_003027.1); fowlpox virus (NC_002188.1); rabbit fibroma virus (NC_001266.1); Molluscum contagiosum virus (NC_001731.1). The newly described seal parapoxvirus sequence is highlighted in red.

**Supplementary Tables:**

**Supplementary Table S1: Reads produced by Oxford Nanopore sequencing.**

|  | total reads | long reads (>3,000bp) | mapped to SePPV^1^ |
| --- | --- | --- | --- |
| template | 147,875 | 10,857 | 23 |
| complement | 73,237 | 5,450 | 14 |
| 2D | 58,642 | 4,210 | 11 |
| ^1^ Oxford Nanopore long reads were aligned to the SePPV genome using LAST and filtered by a minimum alignment length of 3000 base pairs as described in the material and methods section. | | | |

**Supplementary Table S2: ORFs predicted for ORFV but not represented in Seal parapoxvirus.**

| ORF | predicted function in ORFV^1^ |  |
| --- | --- | --- |
| 001 | hypothetical protein |  |
| 002 | hypothetical protein |  |
| 005 | hypothetical protein |  |
| 012 | hypothetical protein |  |
| 013 | hypothetical protein |  |
| 031 | hypothetical protein |  |
| 033 | putative IMV membrane protein |  |
| 089 | virion membrane protein |  |
| 091 | putative IMV membrane protein |  |
| 107 | virion morphogenesis |  |
| 111 | hypothetical protein |  |
| 116 | hypothetical protein |  |
| 117 | GM-CSF/IL-2 inhibition factor like protein |  |
| 120 | hypothetical protein |  |
| 126 | ankyrin repeat protein |  |
| 128 | ankyrin repeat protein |  |
| ^1^ AY386264.1. | | |

**Supplementary Datasets:**

**Dataset S1: Sequence homology between seal parapoxvirus open reading frame translation products and proteins encoded by 43 RefSeq genomes of poxviridae family members.**

Translation products from 120 open reading frames of seal parapoxvirus (left columns) were aligned to proteins encoded by all annotated coding sequences (CDS) of 43 fully sequenced poxvirus genomes deposited in the RefSeq database as of 27/02/2017. Alignments were carried out using the blastp tool from the NCBI blast+ package (v2.6.0) using default stringency parameters and retaining all alignments covering at least 30% of the query and subject sequences. The right columns provide details the most significant alignment observed for each individual translation product, with blank fields indicating that no significant match was observed.

Columns headers indicate the following: ***ORF****:* identifier (locus_tag field) of individual ORFs/CDS regions; *start* position, *end* position and *strand* information are given in bed file format: ***start:*** start position of the ORF; ***end:*** end position of the ORF (including stop codon); ***strand:*** coding strand; ***product length:*** expected length, in amino acids, of the encoded translation product; ***predicted product function:*** predicted function of proteins encoded by seal parapoxvirus, based on annotated functions of aligned poxvirus proteins; ***species id:*** definition and accession of the RefSeq genome encoding the aligned protein; ***% aligned query/subject***: percentage of the translation products from seal parapoxvirus (query) or the RefSeq genome (subject) covered by the alignment; ***% identity/similarity***: percentage of identical or similar amino acid matches, respectively, in the alignment.

The accession numbers of the 43 poxvirus genomes used for the alignments were: NC_032111, NC_005337, NC_003391, NC_005309, NC_003663, NC_006966, NC_004105, NC_002188, NC_004003, NC_003027, NC_001731, NC_003310, NC_001132, NC_008030, NC_005336, NC_025963, NC_024446, NC_024447, NC_013804, NC_030656, NC_001266, NC_027213, NC_004002, NC_031038, NC_022563, NC_003389, NC_008291, NC_028238, NC_006998, NC_001611, NC_031033, NC_005179, NC_002642, NC_015960, NC_021247, NC_002520, NC_023426, NC_021248, NC_021249, NC_001993, NC_021246, NC_016924, NC_027707

**Dataset S2: Sequence homology between seal parapoxvirus open reading frame translation products and proteins encoded by pseudocowpox virus or vaccinia virus.**

Translation products from 120 open reading frames of seal parapoxvirus (left columns) were aligned to proteins encoded by annotated coding sequences (CDS) of pseudocowpox virus (NC_013804, center columns) or vaccinia virus (NC_006998, right columns). The table lists only seal parapoxvirus open reading frames producing significant alignments to annotated translation products from both reference genomes. See footnotes to dataset S1 for details regarding homology searches and column header info.
